# Resonance Fluorescence from Waveguide-Coupled, Strain-Localized, Two-Dimensional Quantum Emitters

**DOI:** 10.1021/acsphotonics.0c01653

**Published:** 2021-04-09

**Authors:** Carlos Errando-Herranz, Eva Schöll, Raphaël Picard, Micaela Laini, Samuel Gyger, Ali W. Elshaari, Art Branny, Ulrika Wennberg, Sebastien Barbat, Thibaut Renaud, Marc Sartison, Mauro Brotons-Gisbert, Cristian Bonato, Brian D. Gerardot, Val Zwiller, Klaus D. Jöns

**Affiliations:** † Department of Applied Physics, KTH Royal Institute of Technology, 114 28 Stockholm, Sweden; ‡ Department of Physics, Paderborn University, 33098 Paderborn, Germany; ∥ Institute for Photonics and Quantum Sciences, SUPA, Heriot-Watt University, Edinburgh EH14 4AS, United Kingdom

**Keywords:** two-dimensional materials, single-photon emitter, photonic integrated circuit, quantum photonics, resonance fluorescence, strain engineering

## Abstract

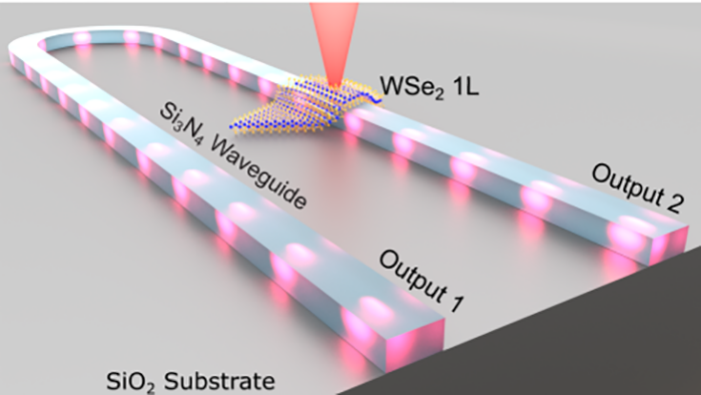

Efficient on-chip integration of single-photon emitters imposes a major bottleneck for applications of photonic integrated circuits in quantum technologies. Resonantly excited solid-state emitters are emerging as near-optimal quantum light sources, if not for the lack of scalability of current devices. Current integration approaches rely on cost-inefficient individual emitter placement in photonic integrated circuits, rendering applications impossible. A promising scalable platform is based on two-dimensional (2D) semiconductors. However, resonant excitation and single-photon emission of waveguide-coupled 2D emitters have proven to be elusive. Here, we show a scalable approach using a silicon nitride photonic waveguide to simultaneously strain-localize single-photon emitters from a tungsten diselenide (WSe_2_) monolayer and to couple them into a waveguide mode. We demonstrate the guiding of single photons in the photonic circuit by measuring second-order autocorrelation of g^(2)^(0) = 0.150 ± 0.093 and perform on-chip resonant excitation, yielding a g^(2)^(0) = 0.377 ± 0.081. Our results are an important step to enable coherent control of quantum states and multiplexing of high-quality single photons in a scalable photonic quantum circuit.

Large-scale on-chip quantum technologies are crucial to realize the long-standing goals of photonic quantum information processing, such as quantum communication,^1^ quantum simulation,^2^ and quantum computing based on cluster-state generation.^3,4^ A promising route toward large-scale quantum information processing relies on single-photon qubits and is based on quantum emitters, memories, and detectors interconnected via photonic integrated circuits (PICs) .^5^

Single-photon emitter integration into PICs has been achieved by embedding quantum dots into III–V PIC platforms,^6^ with limited scalability due to their optical loss, large waveguide bend radius, and low fabrication yields. To utilize the scaling offered by PICs, pick-and-place techniques have been developed to integrate III–V semiconductor quantum dots,^7^ diamond color centers,^8^ and organic molecules^9,10^ into silicon (Si) and silicon nitride (SiN) waveguide platforms. A drawback of this approach lies on the stringent requirements for emitter fabrication and the precise pick-and-place of individual emitters, which drastically limit the scalability of this technology.

A promising candidate to overcome the current scalability limitations of quantum PICs is based on two-dimensional (2D) materials.^11^ In particular, transition metal dichalcogenides (TMDs)^12−17^ enable hundreds of single-photon emitters by a single pick-and-place transfer using localized strain.^18,19^ While the precise origin of these emitters is under debate, a microscopic model that predicts many experimentally observed physical features (such as the g-factors, fine-structure splitting, radiative lifetimes, and polarization dependence) for WSe_2_ single-photon emitters is that of intervalley defect excitons arising from the hybridization of the dark excitons, modulated in energy by local strain, and defect states associated with intrinsic Se vacancies.^20^ Efforts toward 2D TMD single-photon emitter integration into PICs are on the rise, including the transfer of tungsten diselenide (WSe_2_)^21^ and molybdenum disulfide (MoS_2_)^22^ monolayers onto SiN cavities, and WSe_2_ monolayer single-photon emitters on the facet of a titanium-indiffused lithium niobate waveguide with a large mode size,^23^ and on top of a lossy plasmonic slot waveguide.^24^ More scalable approaches have been initiated, such as the coupling of single photons from a 90 nm thick gallium selenide layered semiconductor in SiN waveguides,^25^ photoluminescence from a WSe_2_ monolayer into a SiN waveguide,^26^ and emission from hexagonal boron nitride (hBN) in an aluminum nitride waveguide.^27^ However, single-photon emission into a photonic circuit from deterministic strain-localized quantum emitters has proven to be elusive, let alone resonant excitation of 2D quantum emitters through a PIC, a prerequisite for future initialization and coherent control of quantum states^28^ and for the generation of highly indistinguishable photons.^29^

Here, we address these challenges by (i) inducing strain-localized quantum emitters at the waveguide edges, (ii) multiplexing emitters into the same waveguide mode, (iii) demonstrating waveguide-coupled single-photon emission, and (iv) performing resonant excitation of a single quantum emitter through the waveguide. Our results show the potential of combining 2D semiconductors with PICs toward large-scale quantum technologies by realizing crucial building blocks for future complex circuits.

## Results

### Strain-Localized Emitters in a 2D Semiconductor

**Figure 1:**
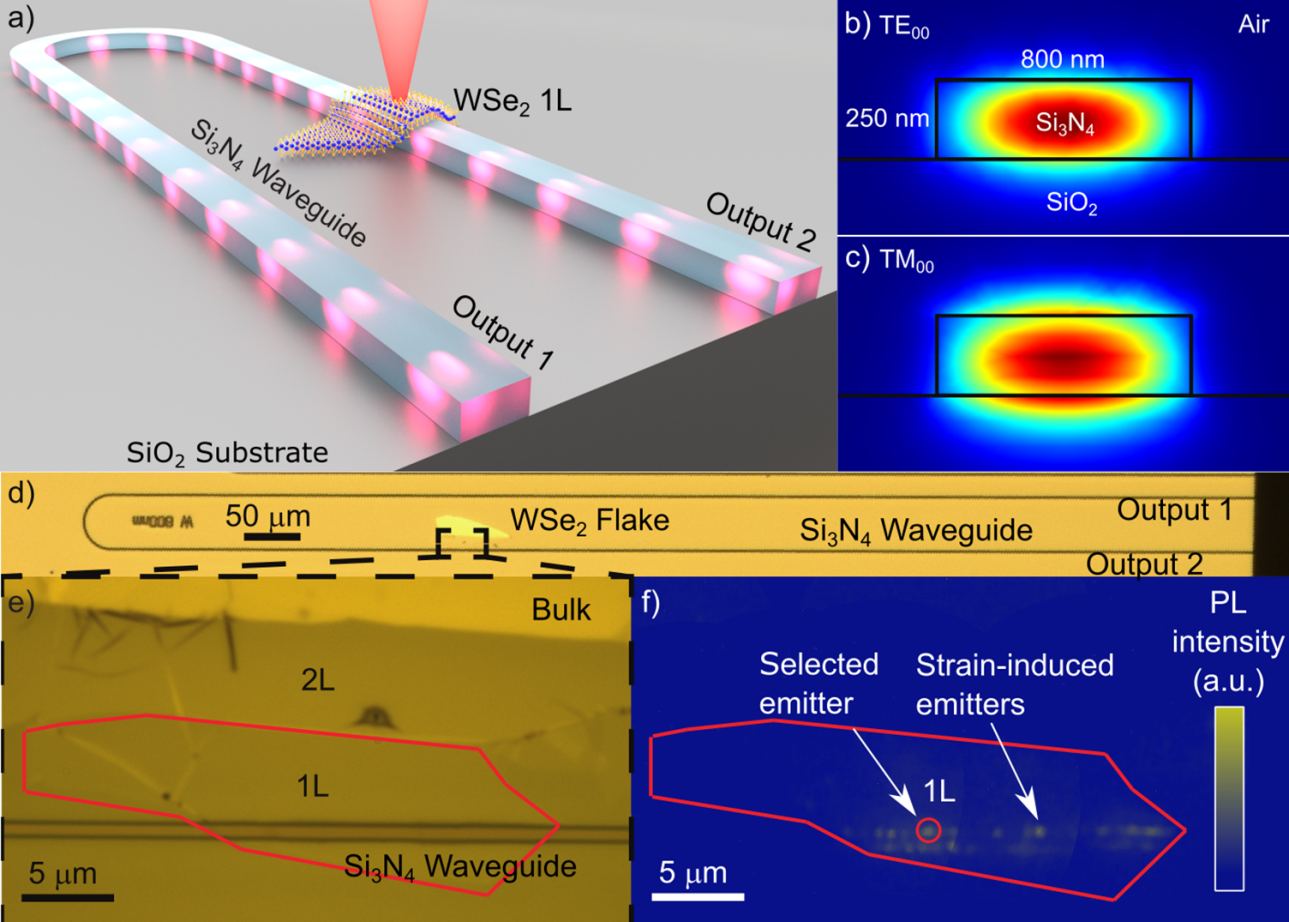
(a) Artistic illustration of the coupled WSe_2_ monolayer (1L) single-photon emitter and the Si_3_N_4_ waveguide. (b) Finite element method eigenmode simulation of the fundamental quasi-TE and (c) quasi-TM waveguide modes at 770 nm wavelength. (d) Microscope image of the Si_3_N_4_ waveguide with (e) zoom-in of the WSe_2_ flake. The monolayer is marked in red (1L). (f) Photoluminescence with defocused excitation shows strain-localized emitters along the waveguide edges. Emitter 1 is marked with a red circle.

**Figure 2:**
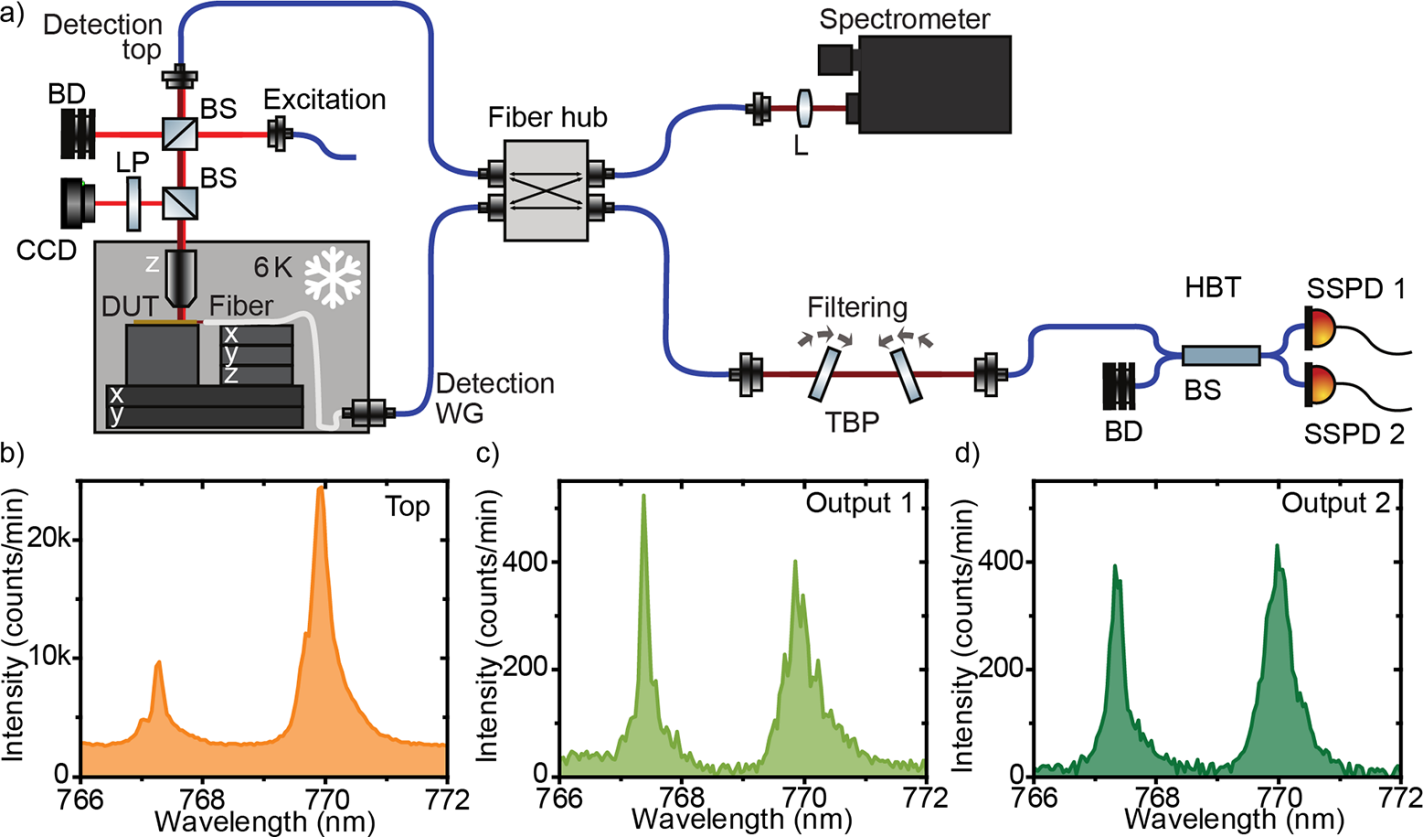
(a) Modular setup consisting of a red laser excitation, a confocal detection path (detection top), and a second detection path from the waveguide facet through a lensed fiber (detection WG). In the fiber hub, the signals can be routed to the spectrometer or the Hanbury Brown and Twiss setup (HBT), which includes a free-space filtering by two tunable bandpass filters (TBP). DUT, device under test; BS, beam splitter; LP, long-pass filter; L, lens; BD, beam dump; SSPD, superconducting single-photon detector. (b) Spectra from emitter 1 at 770 nm taken from top and (c) through the waveguide from output 1 and (d) from output 2.

Figure 1a shows a schematic of our sample, consisting of a U-shaped Si_3_N_4_ waveguide on a SiO_2_ bottom cladding. The designed waveguide geometry supports the fundamental quasi-TE and quasi-TM waveguide modes, as shown in Figure 1b,c. The microscope image in Figure 1d gives an overview of the whole structure, with cleaved facets and an exfoliated WSe_2_ monolayer (1L) placed on top of the waveguide using a dry-transfer method^30^ (see Figure 1e and Supporting Information: Sample geometry and fabrication). Figure 1f shows photoluminescence from emitters in the sample under defocused excitation, recorded using a CCD camera with a 700 nm long pass filter to remove backscattered laser light. The measurements were performed with a modular setup consisting of a closed-cycle cryostat at 6 K, where the sample was placed on a piezoelectric movable stage, a spectrometer, and a Hanbury Brown and Twiss (HBT) second-order correlation measurement setup, as shown in Figure 2a. A detailed description of the setup is given in the Supporting Information: Experimental setup for top excitation. The emitters in the monolayer were excited from the top through a microscope objective with a red pulsed laser (638 nm) with a variable repetition rate of 5–80 MHz. In line with reported strain-localization of single-photon emitters,^18,19^ we observe two lines of spatially localized emitters along the waveguide edges. We observe wrinkles crossing the waveguide in most of the emitter locations (see Figure S1 for SEM images), while we do not observe any photoluminescence other than in the edges of the waveguide. This leads us to believe that the two-dimensional strain localization arose from monolayer wrinkles perpendicularly crossing the waveguide edge (see Supporting Information: Sample geometry and fabrication for further elaboration). This way we are able to multiplex several emitters into the same waveguide mode by a single transfer step (see Supporting Information: Multiplexed emitters in the waveguide for more waveguide-coupled emitters).

By focusing the excitation laser onto the sample and using a confocal microscopy setup, we recorded the photoluminescence spectra of single emitters. Figure 2b shows the spectrum of an emitter marked in Figure 1f collected out of plane of the waveguide through the objective (detection top). To identify the peaks, we performed polarization-resolved photoluminescence spectroscopy (see Supporting Information: Polarization resolved photoluminescence spectroscopy), which indicates that both lines show a two-level system behavior. Due to the different intensity ratios in the collection out-of-plane and in-plane of the waveguide, we believe that they most likely stem from different emitters, which is possible due to our excitation spot size of approximately 2 μm. Figures 2c,d show the spectra at the same location collected through the two waveguide output ports. The line at 770 nm, emitter 1, is used for all further measurements under nonresonant excitation.

**Figure 3:**
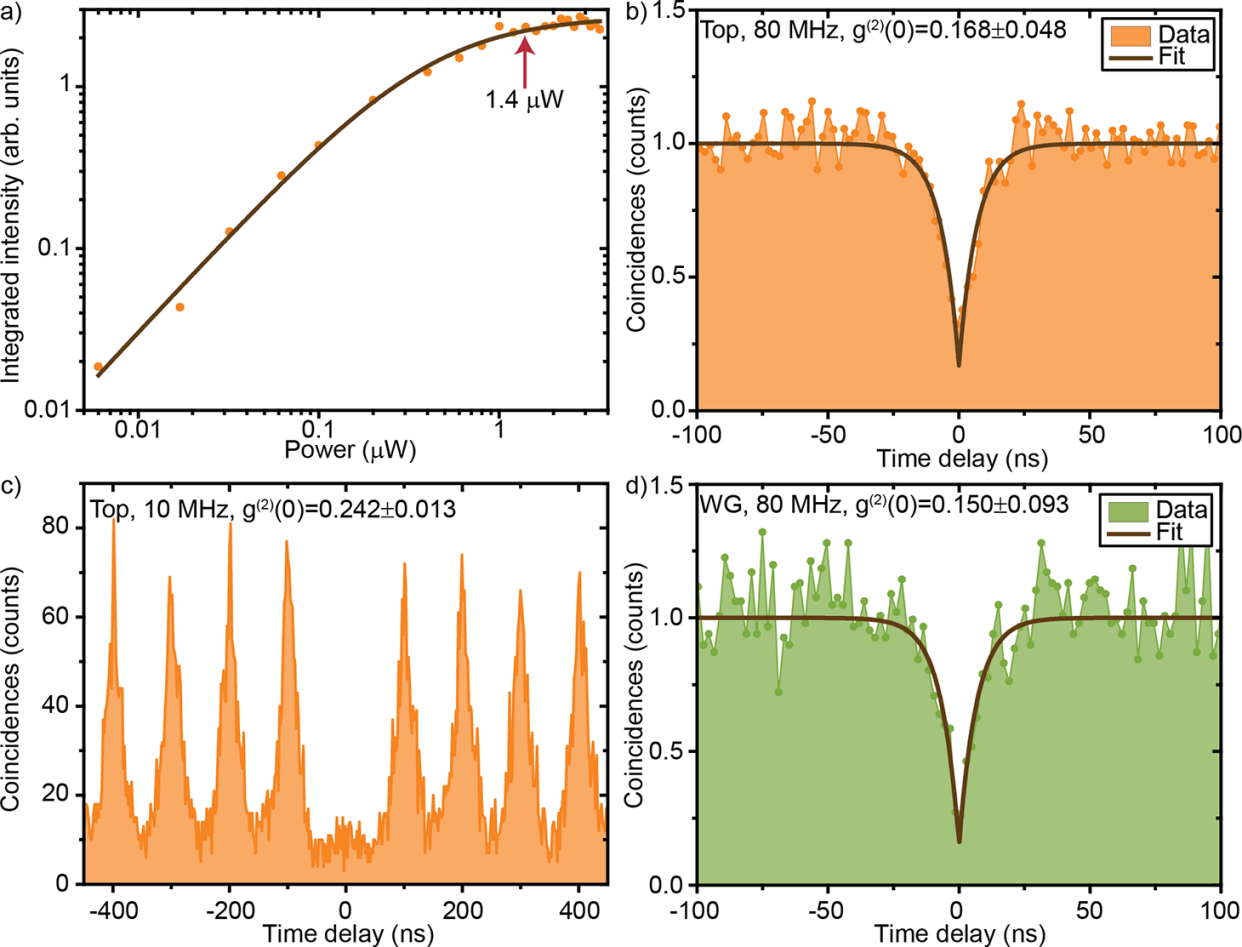
(a) Power series for emitter 1 with a repetition rate of 80 MHz. For all correlation measurements, the emitter was excited with 1.4 μW, that is, at the start of the saturation plateau. (b) Second-order autocorrelation measurement from the top, (c) with a lower repetition rate (10 MHz), and (d) through the waveguide output 1.

A common signature of a two-level system is saturation of the emission intensity with increasing excitation power, shown in Figure 3a in a double-logarithmic plot. Fitting the data as described in the Supporting Information: Power-dependent photoluminescence measurements, we extracted a saturation power of 414 ± 48 nW, corresponding to 3392 counts/s on the single-photon detector. All further measurements were performed with an excitation power of 1.4 μW, located at the start of the saturation plateau for a best trade-off between high-emission intensity and increasing background.

### Single-Photon Emission from a 2D Emitter

To confirm single-photon emission, we performed a second-order autocorrelation measurement on emitter 1, filtered by two overlapping tunable bandpass filters (bandwidth 20 nm) and with a time binning of 2048 ps. Although the emitter was excited with a 80 MHz repetition rate pulsed laser, our second-order autocorrelation measurement, shown in Figure 3b, resembles a measurement under a continuous-wave laser excitation. We investigated this by measuring the emission lifetime with a lower laser repetition rate of 5 MHz (see Supporting Information: Lifetime measurement). Fitting the data with a double-exponential decay, we extracted a lifetime of 18.3 ± 1 ns, which is significantly longer than the separation of two consecutive excitation pulses of 12.5 ns corresponding to a repetition rate of 80 MHz. This in turns leads to a strong overlap between neighboring peaks in the histogram, which can not be distinguished from the noise on the Poisson level. Our simulation results (see Supporting Information: Analysis of second-order autocorrelation measurements under nonresonant excitation) suggest that, under this circumstance, the pulsed second-order autocorrelation measurement can be treated like a continuous-wave measurement. Fitting the data with the formula given in the Supporting Information yields a g^(2)^(0) of 0.168 ± 0.048, well below 0.5 (see Figure 3b), which demonstrates the single-photon nature of the light emission from our 2D emitter. Additionally, we measured the second-order autocorrelation with a repetition rate of 10 MHz shown in Figure 3c, yielding a g^(2)^(0) of 0.242 ± 0.013 without post-selection (see Supporting Information for the analysis).

### Single-Photons from a 2D Emitter through a Si_3_N_4_ Waveguide

Next, we investigated waveguide coupling of single-photon emission from 2D WSe_2_ emitters. We simulate the coupling efficiency from the emitter, approximated by a planar dipole, into the waveguide modes (see Supporting Information: Waveguide coupling simulations). By varying the dipole orientation and positions along the top edge of the waveguide, we calculated its emission into the fundamental quasi-TE (TE00) and quasi-TM (TM00) waveguide modes. The unidirectional coupling efficiency to the fundamental modes when the dipole is located at the edge of the waveguide is, on average, for all possible in-plane dipole orientations, 0.32% and 0.34% to the TE00 and TM00 mode, respectively. Experimentally, we collect the waveguide-coupled emission using a lensed single-mode fiber mounted on an adjacent, independently movable piezoelectric stage, and aligned to one of the waveguide ends. For all waveguide coupled measurements, the fiber was coupled to output 1, marked in Figure 1a. We performed a second-order autocorrelation measurement through the waveguide, shown in Figure 3d, yielding g^(2)^(0) = 0.150 ± 0.093. This value shows no degradation with respect to the free-space *g*^(2)^(0) value, and demonstrates strain-localized single-photon emission into a waveguide.

In the current design, the emitters are located at the edge of the waveguide, yielding sub-optimal coupling efficiency to the fundamental waveguide modes (see simulations in the Supporting Information: Waveguide coupling simulations). More efficient coupling can be achieved by localizing the emitter centered at the top of the waveguide, with an average directional coupling efficiency of 2.48% and 3.01% in the TE00 and TM00 modes (see Supporting Information) or by encapsulation of the emitter, with up to 22% directional coupling for TE00. The compatibility between the localization scheme and the optimal PIC geometry demands nontrivial solutions, which currently stand as remaining challenges hindering efficient coupling. A solution may involve inducing emitters with a helium-focused ion beam^31^ or point-localizing emitters using the strain arising from pillars, gaps, and terminations along waveguides. Alternative methods might be the use of cavities or Moiré-trapped excitons.^32−35^

### Resonance Fluorescence of Waveguide-Coupled 2D Quantum Emitters

Finally, we used our integrated device to perform resonant excitation using side-excitation^36^ through the waveguide output 1. So far, only off-chip confocal resonant excitation of WSe_2_ and hBN emitters have been reported, requiring data postprocessing by either postselection of time intervals when the emitter was on resonance to combat spectral wandering^37^ or laser background subtraction.^38^ Another approach has been to spectrally filter the zero-phonon line together with the resonant laser and only collecting the phonon sideband.^39^

**Figure 4:**
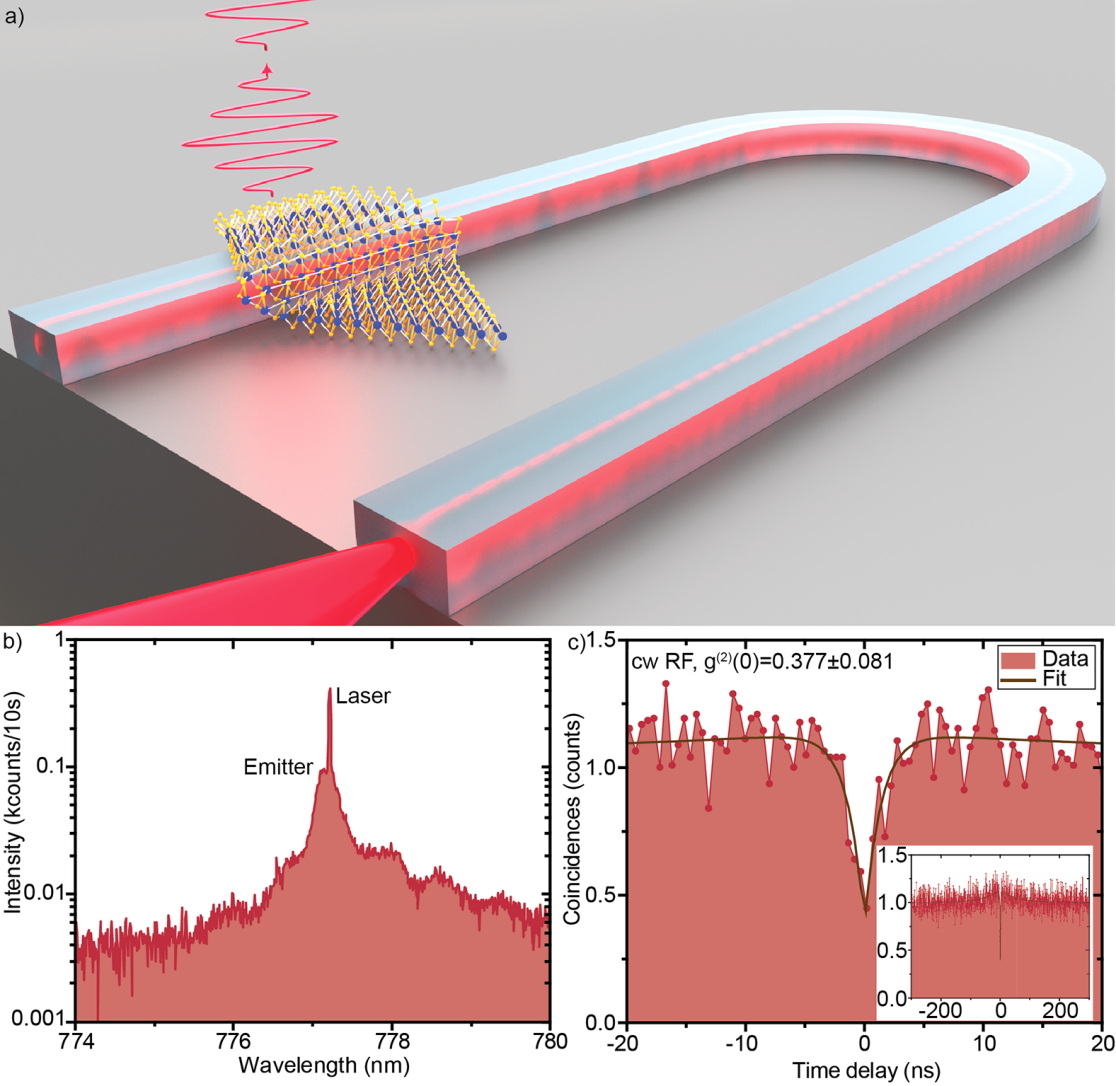
(a) Artistic illustration of the coupled WSe_2_ monolayer single-photon emitter on the Si_3_N_4_ waveguide. The 2D emitter is excited with a continuous-wave (cw) laser coupled to the waveguide. The emitted signal is detected from the top through a microscope objective. (b) Resonance fluorescence (RF) spectrum of emitter 2 and residual laser in a semilogarithmic plot. (c) Second-order autocorrelation measurement under resonant excitation through the waveguide and detection from the top showing clear single-photon emission. Inset: Same measurement for a longer time window showing bunching originating from spectral diffusion.

Here, we achieve sufficient laser suppression in our waveguide-coupled circuit to measure the second-order correlation function without the need of background subtraction nor complex post-processing analysis. Instead, we perform on-the-fly optimization of polarization suppression and only stop and restart the measurement for realignment if the on-the-fly suppression malfunctions. Our resonant excitation and detection scheme of a waveguide-coupled 2D quantum emitter is artistically illustrated in Figure 4a. A continuous-wave diode laser with a linewidth of 50 kHz is coupled via a lensed fiber into the waveguide, which guides the excitation light to the monolayer. The emitted signal is collected from the top by a microscope objective. In this scheme, a large portion of the laser remains in the waveguide, and only the light scattered by the waveguide surface is collected by the objective. Further spatial suppression of laser light is achieved by fiber coupling the collected signal. To distinguish the resonance fluorescence signal from the remaining spatially overlapping scattered laser light, the laser is suppressed in a polarization suppression setup (see Supporting Information: Methods for second-order autocorrelation measurements under resonant excitation for a detailed description). The resonance fluorescence of emitter 2 as well as the remaining laser light are shown in Figure 4b, with deliberately nonoptimal laser suppression for visualization. This shows the slight mismatch between the laser wavelength and the emitter spectrum, which stems from a spectral shift of the emitter when the laser is on-resonance. We then performed a second-order autocorrelation measurement with a time binning of 512 ps (see Supporting Information for more details), shown in Figure 4c, yielding a g^(2)^(0) = 0.377 ± 0.081 without postprocessing of the data, such as laser background subtraction, indicating clear single-photon emission from the emitter under pure resonant excitation. The nonzero value at zero time delay stems mainly from remaining laser scattering. Furthermore, the emitter shows light bunching on the time scale of 50 ns (inset of Figure 4c), originating from spectral diffusion.

The limited single-photon characteristics under resonant excitation stem mainly from the remaining scattered laser that can not be suppressed in the polarization suppression setup. Due to the current setup and device, the polarization of the input laser as well as the scattered laser from the waveguide can not be well controlled and was therefore not perfectly suppressed. These challenges can be overcome by using polarization-maintaining fibers, free-space coupling of the laser to the waveguide, on-chip polarization or mode control, or time gating of the signal using fast electronics. Additionally, several groups^40,41^ have reported off-chip resonant excitation of waveguide-coupled quantum emitters without the need for polarization suppression, taking advantage of high-quality waveguides with smooth sidewalls. Next steps toward coherent control of PIC-coupled 2D quantum emitters will require on-chip resonance fluorescence by excitation and detection through waveguides and pulsed resonant excitation. The significant spectral jitter of these emitters makes further measurements that exploit the benefits of resonant excitation, such as two-photon interference, challenging. Further progress will likely require the use of cavities, encapsulation, and control of the Fermi level.

## Discussion

Compared to the state of the art,^26^ our results demonstrate the following: (1) Clear localization of emitters in our undamaged monolayer in the strained regions due to the waveguide. (2) A g^(2)^(0) of 0.17 without background subtraction, which clearly demonstrates single-photon emission. This is significantly below the previously reported g^(2)^(0) near 0.5 with background subtraction. (3) Through-waveguide HBT measurement, shown here for the first time, which unambiguously proves the coupling of single-photons into their straining waveguide. (4) Through-waveguide resonance fluorescence excitation.

Our results illustrate a path toward quantum PIC where a single TMD monolayer generates many emitters, which may overcome the current bottlenecks of single-emitter pick-and-place methods. A key application of our technology is large-scale quantum light sources, where a single transfer of a TMD monolayer creates an array of localized emitters efficiently coupled via on-chip cavities into waveguides equipped with reconfigurable on-chip filters such as ring resonators. The output is a large number of indistinguishable single photons, which can be routed into the optical fiber network for quantum communications or remain on-chip for cluster-state quantum computing or simulation. In addition to multiplexing, large-scale quantum photonic circuits require two-photon interference between photons emitted from independent on-chip sources. The different electrostatic and strain environments of each single-photon emitter make emitted photons spectrally different, hampering quantum interference. These spectral differences may be bridged by controlling the strain experienced by the emitter via structural design of the underlying SiN. Fine alignment of emission spectra can then be achieved using active strain^42^ and Stark effect^43^ based tuning.

Quantum photonic integrated circuits provide a scalable and cost-efficient route to increasingly complex quantum systems, and constitute an enabling platform for applications such as quantum key distribution, quantum simulation, and cluster-state quantum computing. We have developed a hybrid deterministic integration method of single-photon emitters in 2D materials into silicon-based photonic circuits by exploiting the creation of strain-localized quantum emitters at the edges of a photonic waveguide. Our proof-of-principle structure maintains a single-photon purity of 0.150 ± 0.093 and resonance fluorescence with g^(2)^(0) = 0.377 ± 0.081. These experimental results and proposed designs provide a hybrid integration platform with promising scaling prospects, crucial for large-scale quantum integrated circuits.

## Supporting Information Available

Sample geometry and fabrication; Experimental setup for top excitation; Multiplexed emitters in the waveguide; Polarization-resolved photoluminescence spectroscopy; Power-dependent photoluminescence measurements; Lifetime measurement; Analysis of second-order autocorrelation measurements under nonresonant excitation; Waveguide coupling simulations; Methods for second-order autocorrelation measurements under resonant excitation (PDF)

## References

[ref1] Borregaard J.; Pichler H.; Schröder T.; Lukin M. D.; Lodahl P.; Sørensen A. S. One-Way Quantum Repeater Based on near-Deterministic Photon-Emitter Interfaces. Phys. Rev. X 2020, 10, na 10.1103/PhysRevX.10.021071.

[ref2] Aspuru-Guzik A.; Walther P. Photonic Quantum Simulators. Nat. Phys. 2012, 8, 285–291. 10.1038/nphys2253.

[ref3] Rudolph T. Why I Am Optimistic about the Silicon-Photonic Route to Quantum Computing. APL Photonics 2017, 2, 030901 10.1063/1.4976737.

[ref4] Ladd T. D.; Jelezko F.; Laflamme R.; Nakamura Y.; Monroe C.; O’Brien J. L. Quantum Computers. Nature 2010, 464, 45–53. 10.1038/nature08812. 20203602

[ref5] Flamini F.; Spagnolo N.; Sciarrino F. Photonic Quantum Information Processing: A Review. Rep. Prog. Phys. 2019, 82, 016001 10.1088/1361-6633/aad5b2. 30421725

[ref6] Hepp S.; Jetter M.; Portalupi S. L.; Michler P. Semiconductor Quantum Dots for Integrated Quantum Photonics (Adv. Quantum Technol. 9/2019). Advanced Quantum Technologies 2019, 2, 1970053 10.1002/qute.201970053.

[ref7] Kim J.-H.; Aghaeimeibodi S.; Carolan J.; Englund D.; Waks E. Hybrid Integration Methods for On-Chip Quantum Photonics. Optica 2020, 7, 291–308. 10.1364/OPTICA.384118.

[ref8] Mouradian S. L.; Schröder T.; Poitras C. B.; Li L.; Goldstein J.; Chen E. H.; Walsh M.; Cardenas J.; Markham M. L.; Twitchen D. J.; Lipson M.; Englund D. Scalable Integration of Long-Lived Quantum Memories into a Photonic Circuit. Phys. Rev. X 2015, 5, 031009 10.1103/PhysRevX.5.031009.

[ref9] Colautti M.; Lombardi P.; Trapuzzano M.; Piccioli F. S.; Pazzagli S.; Tiribilli B.; Nocentini S.; Cataliotti F. S.; Wiersma D. S.; Toninelli C. A 3D Polymeric Platform for Photonic Quantum Technologies. Advanced Quantum Technologies 2020, 3, 2000004 10.1002/qute.202000004.

[ref10] Lombardi P.; Ovvyan A. P.; Pazzagli S.; Mazzamuto G.; Kewes G.; Neitzke O.; Gruhler N.; Benson O.; Pernice W. H. P.; Cataliotti F. S.; Toninelli C. Photostable Molecules on Chip: Integrated Sources of Nonclassical Light. ACS Photonics 2018, 5, 126–132. 10.1021/acsphotonics.7b00521.

[ref11] Stanford M. G.; Rack P. D.; Jariwala D. Emerging Nanofabrication and Quantum Confinement Techniques for 2D Materials beyond Graphene. npj 2D Materials and Applications 2018, 2, 1–15. 10.1038/s41699-018-0065-3.

[ref12] Tonndorf P.; Schmidt R.; Schneider R.; Kern J.; Buscema M.; Steele G. A.; Castellanos-Gomez A.; van der Zant H. S. J.; Michaelis de Vasconcellos S.; Bratschitsch R. Single-Photon Emission from Localized Excitons in an Atomically Thin Semiconductor. Optica 2015, 2, 347–352. 10.1364/OPTICA.2.000347.

[ref13] Kumar S.; Kaczmarczyk A.; Gerardot B. D. Strain-Induced Spatial and Spectral Isolation of Quantum Emitters in Mono- and Bilayer WSe2. Nano Lett. 2015, 15, 7567–7573. 10.1021/acs.nanolett.5b03312. 26480237 PMC4643357

[ref14] Srivastava A.; Sidler M.; Allain A. V.; Lembke D. S.; Kis A.; Imamoğlu A. Optically Active Quantum Dots in Monolayer WSe_2_. Nat. Nanotechnol. 2015, 10, 491–496. 10.1038/nnano.2015.60. 25938570

[ref15] He Y.-M.; Clark G.; Schaibley J. R.; He Y.; Chen M.-C.; Wei Y.-J.; Ding X.; Zhang Q.; Yao W.; Xu X.; Lu C.-Y.; Pan J.-W. Single Quantum Emitters in Monolayer Semiconductors. Nat. Nanotechnol. 2015, 10, 497–502. 10.1038/nnano.2015.75. 25938571

[ref16] Koperski M.; Nogajewski K.; Arora A.; Cherkez V.; Mallet P.; Veuillen J.-Y.; Marcus J.; Kossacki P.; Potemski M. Single Photon Emitters in Exfoliated WSe_2_ Structures. Nat. Nanotechnol. 2015, 10, 503–506. 10.1038/nnano.2015.67. 25938573

[ref17] Chakraborty C.; Kinnischtzke L.; Goodfellow K. M.; Beams R.; Vamivakas A. N. Voltage-Controlled Quantum Light from an Atomically Thin Semiconductor. Nat. Nanotechnol. 2015, 10, 507–511. 10.1038/nnano.2015.79. 25938569

[ref18] Branny A.; Kumar S.; Proux R.; Gerardot B. D. Deterministic Strain-Induced Arrays of Quantum Emitters in a Two-Dimensional Semiconductor. Nat. Commun. 2017, 8, 1–7. 10.1038/ncomms15053. 28530219 PMC5458118

[ref19] Palacios-Berraquero C.; Kara D. M.; Montblanch A. R.-P.; Barbone M.; Latawiec P.; Yoon D.; Ott A. K.; Loncar M.; Ferrari A. C.; Atatüre M. Large-Scale Quantum-Emitter Arrays in Atomically Thin Semiconductors. Nat. Commun. 2017, 8, 1–6. 10.1038/ncomms15093. 28530249 PMC5458119

[ref20] Linhart L.; Paur M.; Smejkal V.; Burgdörfer J.; Mueller T.; Libisch F. Localized Intervalley Defect Excitons as Single-Photon Emitters in ${\mathrm{WSe}}_{2}$. Phys. Rev. Lett. 2019, 123, 146401 10.1103/PhysRevLett.123.146401. 31702183

[ref21] Fryett T. K.; Chen Y.; Whitehead J.; Peycke Z. M.; Xu X.; Majumdar A. Encapsulated Silicon Nitride Nanobeam Cavity for Hybrid Nanophotonics. ACS Photonics 2018, 5, 2176–2181. 10.1021/acsphotonics.8b00036.

[ref22] Wei G.; Stanev T. K.; Czaplewski D. A.; Jung I. W.; Stern N. P. Silicon-Nitride Photonic Circuits Interfaced with Monolayer MoS2. Appl. Phys. Lett. 2015, 107, 091112 10.1063/1.4929779.

[ref23] White D.; Branny A.; Chapman R. J.; Picard R.; Brotons-Gisbert M.; Boes A.; Peruzzo A.; Bonato C.; Gerardot B. D. Atomically-Thin Quantum Dots Integrated with Lithium Niobate Photonic Chips [Invited]. Opt. Mater. Express 2019, 9, 441–448. 10.1364/OME.9.000441.

[ref24] Blauth M.; Jürgensen M.; Vest G.; Hartwig O.; Prechtl M.; Cerne J.; Finley J. J.; Kaniber M. Coupling Single Photons from Discrete Quantum Emitters in WSe2 to Lithographically Defined Plasmonic Slot Waveguides. Nano Lett. 2018, 18, 6812–6819. 10.1021/acs.nanolett.8b02687. 30153417

[ref25] Tonndorf P.; Del Pozo-Zamudio O.; Gruhler N.; Kern J.; Schmidt R.; Dmitriev A. I.; Bakhtinov A. P.; Tartakovskii A. I.; Pernice W.; Michaelis de Vasconcellos S.; Bratschitsch R. On-Chip Waveguide Coupling of a Layered Semiconductor Single-Photon Source. Nano Lett. 2017, 17, 5446–5451. 10.1021/acs.nanolett.7b02092. 28796522

[ref26] Peyskens F.; Chakraborty C.; Muneeb M.; Van Thourhout D.; Englund D. Integration of Single Photon Emitters in 2D Layered Materials with a Silicon Nitride Photonic Chip. Nat. Commun. 2019, 10, 1–7. 10.1038/s41467-019-12421-0. 31570712 PMC6768863

[ref27] Kim S.; Duong N. M. H.; Nguyen M.; Lu T.-J.; Kianinia M.; Mendelson N.; Solntsev A.; Bradac C.; Englund D. R.; Aharonovich I. Integrated on Chip Platform with Quantum Emitters in Layered Materials. Adv. Opt. Mater. 2019, 7, 1901132 10.1002/adom.201901132.

[ref28] Warren W. S.; Rabitz H.; Dahleh M. Coherent Control of Quantum Dynamics: The Dream Is Alive. Science 1993, 259, 1581–1589. 10.1126/science.259.5101.1581. 17733021

[ref29] Senellart P.; Solomon G.; White A. High-Performance Semiconductor Quantum-Dot Single-Photon Sources. Nat. Nanotechnol. 2017, 12, 1026–1039. 10.1038/nnano.2017.218. 29109549

[ref30] Castellanos-Gomez A.; Buscema M.; Molenaar R.; Singh V.; Janssen L.; van der Zant H. S. J.; Steele G. A. Deterministic Transfer of Two-Dimensional Materials by All-Dry Viscoelastic Stamping. 2D Mater. 2014, 1, 011002 10.1088/2053-1583/1/1/011002.

[ref31] Klein J.; Lorke M.; Florian M.; Sigger F.; Sigl L.; Rey S.; Wierzbowski J.; Cerne J.; Muller K.; Mitterreiter E.; Zimmermann P.; Taniguchi T.; Watanabe K.; Wurstbauer U.; Kaniber M.; Knap M.; Schmidt R.; Finley J. J.; Holleitner A. W. Site-Selectively Generated Photon Emitters in Monolayer MoS_2_ via Local Helium Ion Irradiation. Nat. Commun. 2019, 10, 1–8. 10.1038/s41467-019-10632-z. 31227692 PMC6588625

[ref32] Tran K.; et al. Evidence for Moiré Excitons in van Der Waals Heterostructures. Nature 2019, 567, 71–75. 10.1038/s41586-019-0975-z. 30804527 PMC11493145

[ref33] Seyler K. L.; Rivera P.; Yu H.; Wilson N. P.; Ray E. L.; Mandrus D. G.; Yan J.; Yao W.; Xu X. Signatures of Moiré-Trapped Valley Excitons in MoSe 2 /WSe 2 Heterobilayers. Nature 2019, 567, 66–70. 10.1038/s41586-019-0957-1. 30804526

[ref34] Brotons-Gisbert M.; Baek H.; Molina-Sanchez A.; Campbell A.; Scerri E.; White D.; Watanabe K.; Taniguchi T.; Bonato C.; Gerardot B. D. Spin-Layer Locking of Interlayer Valley Excitons Trapped in Moire Potentials. Nat. Mater. 2020, 19, 630 10.1038/s41563-020-0687-7. 32451512

[ref35] Baek H.; Brotons-Gisbert M.; Koong Z. X.; Campbell A.; Rambach M.; Watanabe K.; Taniguchi T.; Gerardot B. D. Highly Tunable Quantum Light from Moire Trapped Excitons. Sci. Adv. 2020, 6, eaba8526 10.1126/sciadv.aba8526. 32917702 PMC7486092

[ref36] Muller A.; Flagg E. B.; Bianucci P.; Wang X. Y.; Deppe D. G.; Ma W.; Zhang J.; Salamo G. J.; Xiao M.; Shih C. K. Resonance Fluorescence from a Coherently Driven Semiconductor Quantum Dot in a Cavity. Phys. Rev. Lett. 2007, 99, 187402 10.1103/PhysRevLett.99.187402. 17995437

[ref37] Kumar S.; Brotóns-Gisbert M.; Al-Khuzheyri R.; Branny A.; Ballesteros-Garcia G.; Sánchez-Royo J. F.; Gerardot B. D. Resonant Laser Spectroscopy of Localized Excitons in Monolayer WSe_2_. Optica 2016, 3, 882–886. 10.1364/OPTICA.3.000882.

[ref38] Konthasinghe K.; Chakraborty C.; Mathur N.; Qiu L.; Mukherjee A.; Fuchs G. D.; Vamivakas A. N. Rabi Oscillations and Resonance Fluorescence from a Single Hexagonal Boron Nitride Quantum Emitter. Optica 2019, 6, 542–548. 10.1364/OPTICA.6.000542.

[ref39] Tran T. T.; Kianinia M.; Nguyen M.; Kim S.; Xu Z.-Q.; Kubanek A.; Toth M.; Aharonovich I. Resonant Excitation of Quantum Emitters in Hexagonal Boron Nitride. ACS Photonics 2018, 5, 295–300. 10.1021/acsphotonics.7b00977.

[ref40] Schwartz M.; Rengstl U.; Herzog T.; Paul M.; Kettler J.; Portalupi S. L.; Jetter M.; Michler P. Generation, Guiding and Splitting of Triggered Single Photons from a Resonantly Excited Quantum Dot in a Photonic Circuit. Opt. Express 2016, 24, 3089–3094. 10.1364/OE.24.003089. 26906873

[ref41] Huber T.; Davanco M.; Müller M.; Shuai Y.; Gazzano O.; Solomon G. S. Filter-Free Single-Photon Quantum Dot Resonance Fluorescence in an Integrated Cavity-Waveguide Device. Optica 2020, 7, 380–385. 10.1364/OPTICA.382273.

[ref42] Grosso G.; Moon H.; Lienhard B.; Ali S.; Efetov D. K.; Furchi M. M.; Jarillo-Herrero P.; Ford M. J.; Aharonovich I.; Englund D. Tunable and High-Purity Room Temperature Single-Photon Emission from Atomic Defects in Hexagonal Boron Nitride. Nat. Commun. 2017, 8, 1–8. 10.1038/s41467-017-00810-2. 28951591 PMC5615041

[ref43] Noh G.; Choi D.; Kim J.-H.; Im D.-G.; Kim Y.-H.; Seo H.; Lee J. Stark Tuning of Single-Photon Emitters in Hexagonal Boron Nitride. Nano Lett. 2018, 18, 4710–4715. 10.1021/acs.nanolett.8b01030. 29932664

