# Supplementary material for: Resonance Fluorescence from Waveguide-Coupled, Strain-Localized, Two-Dimensional Quantum Emitters
Source: ACS Photonics. 2021 Apr 9;8(4):1069–76. doi: 10.1021/acsphotonics.0c01653 (PMC8155555; doi:10.1021/acsphotonics.0c01653)
Supplement: Supplementary file 1 — ph0c01653_si_001.pdf [file ph0c01653_si_001.pdf]

Supporting Information:

Resonance fluorescence from waveguide–coupled strain–localized two-dimensional quantum emitters

Carlos Errando-Herranz,[*,†,§] Eva Schöll,[*,†,‡,§] Raphaël Picard,[¶] Micaela Laini,[¶] Samuel Gyger,[†] Ali W. Elshaari,[†] Art Branny,[†] Ulrika Wennberg,[†] Sebastien Barbat,[†] Thibaut Renaud,[†] Marc Sartison,[‡] Mauro Brotons-Gisbert,[¶] Cristian Bonato,[¶] Brian D. Gerardot,[¶] Val Zwiller,[†] and Klaus D. Jöns[*,†,‡]

†*Department of Applied Physics, KTH Royal Institute of Technology, Stockholm, Sweden*

‡*Department of Physics, Paderborn University, 33098 Paderborn, Germany*

¶*Institute for Photonics and Quantum Sciences, SUPA, Heriot-Watt University, Edinburgh, UK*

§*Equal contribution*

E-mail: carloseh@kth.se; eva.schoell@upb.de; klaus.joens@upb.de

This PDF file includes: pages S1-S10, Figs. S1-S8.

Contents

1 Sample geometry and fabrication S-2

2 Experimental setup for top excitation S-3

3 Multiplexed emitters in the waveguide S-3

4 Polarization resolved photoluminescence spectroscopy S-4

5 Power–dependent photoluminescence measurements S-5

6 Lifetime measurement S-5

7 Analysis of second–order autocorrelation measurements under non–resonant excitation S-6

8 Waveguide coupling simulations S-7

9 Methods for second–order autocorrelation measurements under resonant excitation S-7

References S-8

1 Sample geometry and fabrication

The waveguide length is 2.2 mm, with a bend radius of 25 µm, and a cross-section (height × width) of 250 × 800 nm. This waveguide cross-section supports 2 quasi-TE and 2 quasi-TM modes, its fundamental quasi-TE mode index being $n_{TE00} = 1.72$. The flake is positioned at a 750 µm distance from one of the waveguide ends, and covers a waveguide section about 20 µm long.

The sample fabrication started with a 250 nm thin stoichiometric Si_3N_4 film on 3.3 µm SiO_2 on a silicon substrate (Rogue Valley Microdevices). The waveguides were fabricated using electron beam lithography followed by CHF_3-based reactive ion etching, resist stripping, and sample cleaving. Flux zone grown WSe_2 crystals (from 2D semiconductors) were then exfoliated, and monolayers were identified under a microscope and transferred using a polydimethylsiloxane (PDMS) dry stamp process.[S1]

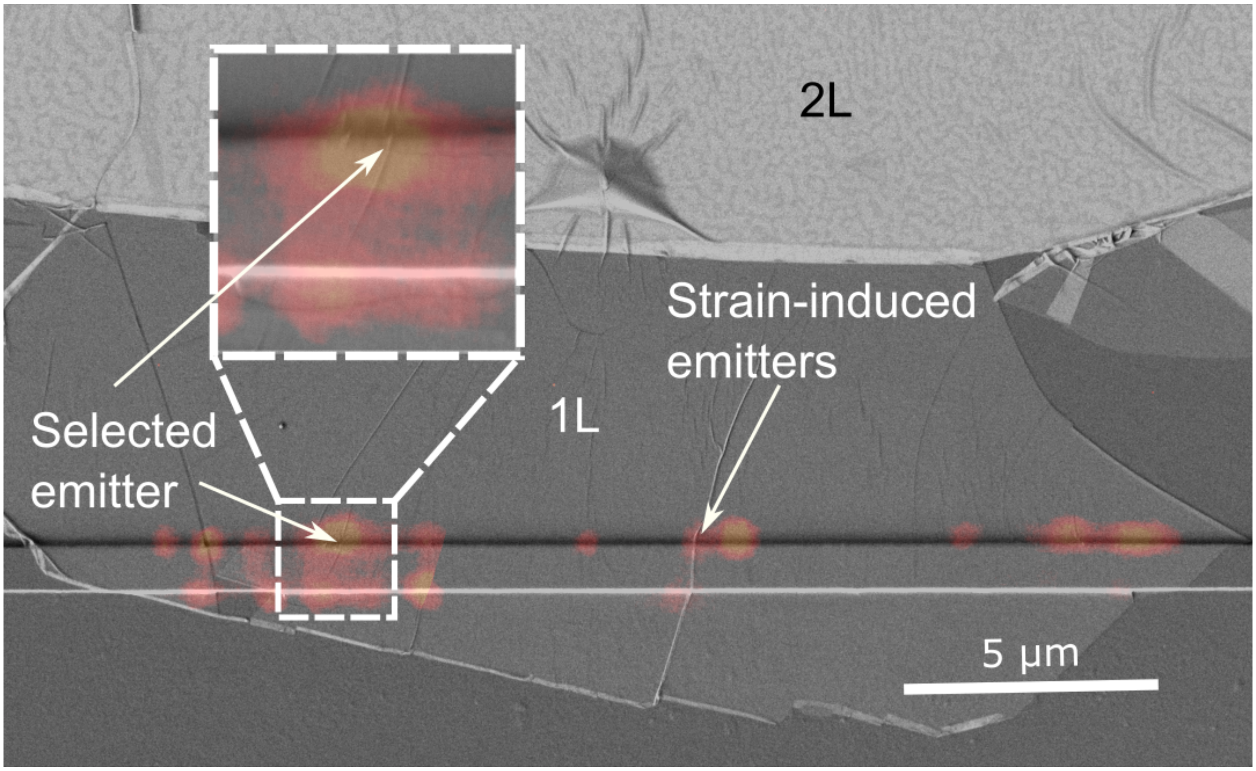

Figure S1: Top SEM images of the sample. The image shows the WSe2 monolayer covering the waveguide, overlapped with our photoluminescence measurement from Fig. 1f. Wrinkles crossing the waveguide are visible. Emitter 1 used for the measurements under non-resonant excitation in the main text is marked accordingly via a white arrow in the inset, and coincides with the position of a wrinkle at the waveguide edge. In the inset we can observe that the emitter matches one of the wrinkles across the waveguide edge.

To confirm that our emitters are strain-induced by the waveguide edges, we acquired scanning electron microscope (SEM) images of the sample. Figure S1 shows the monolayer covering the waveguide with an overlay of the photoluminescence map from Fig.1f in the main manuscript. We see several wrinkles (and one rupture) going across the waveguide.

If emitters were to originate solely from these features, photoluminescence would also be visible across the rest of the flake. Since the emitters only occur on the edges, we conclude that the strain potential inducing the emitters is confined in one dimension by a wrinkle or rupture and in the other dimension by the edge of the waveguide. In the closeup SEM image (Fig. S1) we can observe the wrinkle and waveguide edge inducing the selected emitter E1, used for the non-resonant measurements in the main manuscript.

2 Experimental setup for top excitation

The setup is shown in Fig. 2a in the main text. For all measurements, the sample was placed inside a low–vibration closed–cycle cryostat on piezoelectric xy–positioners and cooled to 6 K. For excitation, a red (638 nm) pulsed laser diode with variable repetition rate of $5 - 80$ MHz was used, which was focused onto the sample using a $50\times, NA = 0.81$ microscope objective. The photoluminescence of the excited emitter was detected in two ways: from the top using free–space optics or through the waveguide. The part of the photoluminescence emitted to the top was collected through the same microscope objective and then coupled into a fiber. The part coupled to the Si_3N_4 waveguide was coupled into a lensed fiber (OZOptics, 780HP, working distance 13 ± 1 µm) positioned near the cleaved facet of one of the waveguide ends. The lensed fiber, which was mounted on an individual xyz–positioner stack, was pre–aligned to the waveguide by sending a narrow linewidth laser at 770 nm through the fiber and maximizing the signal at the other output of the waveguide with the CCD camera through the microscope objective. The fine alignment was done by maximizing the emitter signal on the spectrometer. The fiber–coupled signal was either sent to the CCD of a spectrometer (grating 600 lines/mm) or into a fiber–based Hanbury Brown and Twiss (HBT) type setup to measure the second–order autocorrelation function. This setup consists of a $50 : 50$ fiber beamsplitter connected to two superconducting single–photon detectors (Single Quantum) with efficiencies of 50%, 60%, timing jitters of 20 and 30 ps, and dark count rates of 0.006 and 0.017 cts/s. For all correlation measurements, a single line was filtered from the spectrum using two overlapping free–space tunable bandpass filters with a bandwidth of 20 nm. For excitation with a green laser (532 nm), the excitation was coupled into the setup using a dichroic longpass mirror with the edge at 695 nm (not shown in the setup). To investigate the localization of the emitters in the WSe_2 monolayer, we excited the sample with a de–focused laser (lens with $f = 300$ mm, not shown). The emitted signal was sent onto a CCD camera using a flip–in beamsplitter, and the accompanying backscattered excitation laser was filtered with a 700 nm long pass filter.

3 Multiplexed emitters in the waveguide

Figure S2 shows waveguide–coupled emission from three different 2D emitters, in addition to the two emitters in the main text, strain–localized from a single monolayer transfer.

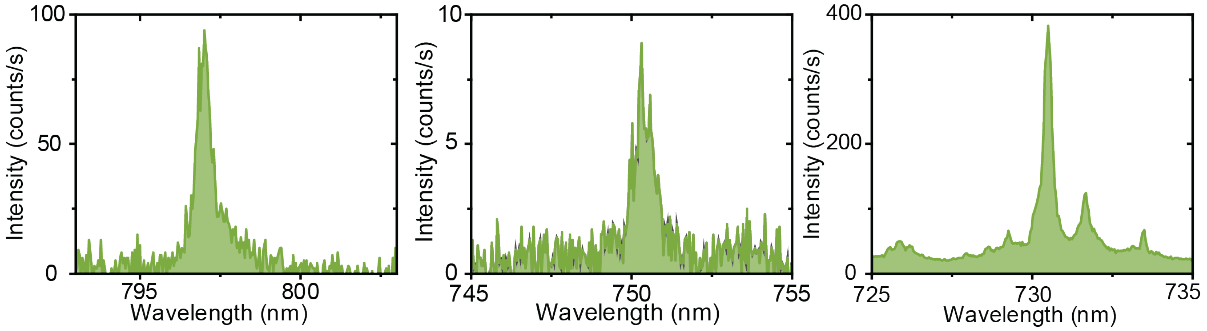

Figure S2: Spectra of 3 waveguide–coupled emitters from the same 2D flake, PL measured through the waveguide. These emitters are in addition to the 2 emitters studied in the main text.

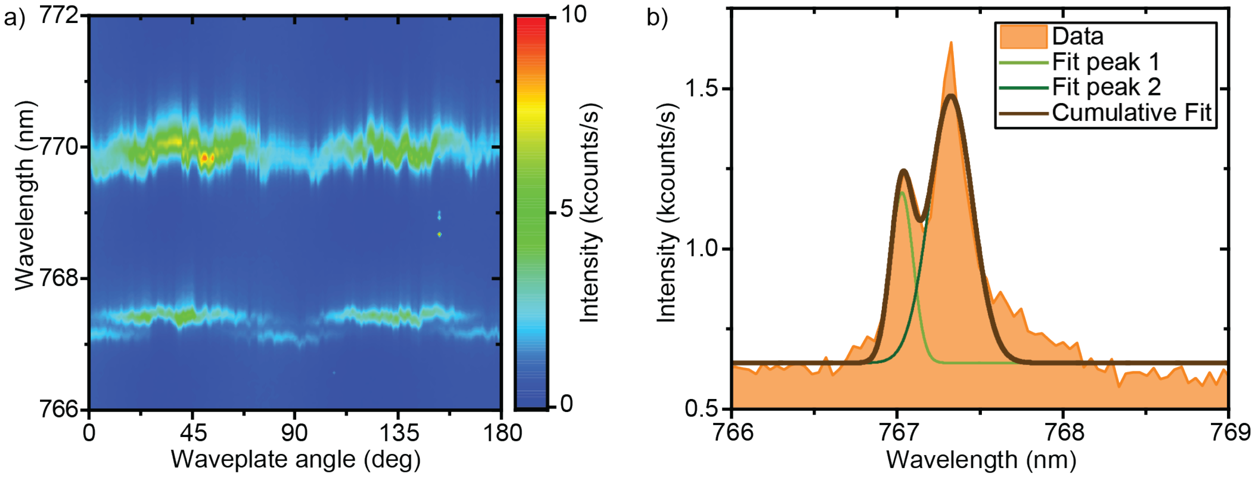

Figure S3: a) Map of emitter 1 under changing half–wave plate angle. b) Spectrum of the line at 767 nm and a halfwave plate angle of 70°. A fit reveals a fine–structure splitting of $624 \pm 31\mu eV$.

4 Polarization resolved photoluminescence spectroscopy

To identify the peaks of the spectra in Fig. 2b-d in the main manuscript, we measured the polarization resolved photoluminescence. We placed a halfwave plate and a fixed linear polarizer in front of the spectrometer and recorded the spectra while automatically rotating the wave plate, as shown in Fig. S3a. Only the line at 767 nm is showing a clear fine–structure splitting. Figure S3b shows the spectrum of this line at a halfwave plate angle of 70°, where both components are well visible. Fitting this data with two Gaussians, reveals a fine–structure splitting of $624 \pm 31\mu eV$, indicating an exciton. The line at 770 nm shows intensity modulation with the same period but without a clear fine structure splitting, which indicates that this line still belongs to an excitonic state where the intensity of one fine–structure component is too dim to see. This leads us to believe that those two lines stem from separate emitters.

5 Power–dependent photoluminescence measurements

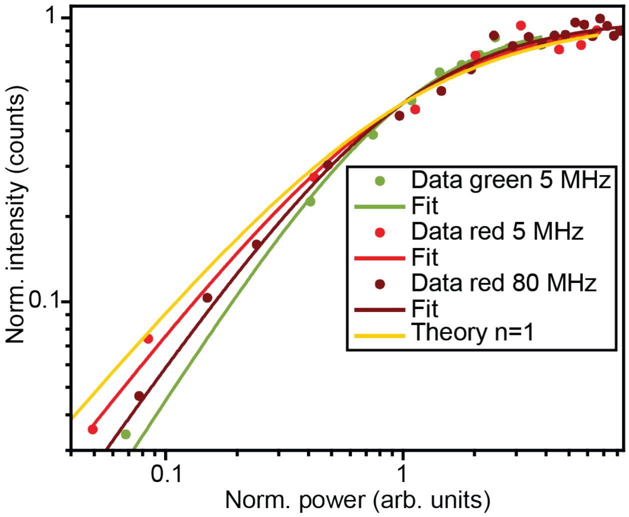

Figure S4: Peak area of emitter 1 as a function of excitation power in a double–logarithmic plot for red (5 and 80 MHz) and green (5 MHz) excitation and the theoretical curve for an one–photon excitation process in yellow.

A non–resonantly driven two–level system saturates with increasing excitation power. We investigated the behaviour for different excitation wavelengths, namely red 638 nm with a repetition rate of 5 and 80 MHz, and green 532 nm with a repetition rate of 5 MHz. In Fig. S4 we show the peak areas as a function of excitation power in a double–logarithmic plot.

All data sets are fitted with $I(P) = I_\infty \frac{\left(\frac{P}{P_{sat}}\right)^n}{\left(\frac{P}{P_{sat}}\right)^n + 1}$ S2 and weighted with $w_i = 1/y_i$ to compensate for the fact of fewer data points at low excitation energies. To compare the data sets, they are normalized with I_∞ and P_{sat}. If the system is excited in a one–photon process, the saturation should follow the formula for $n = 1$ (yellow line in Fig. S4), what is expected since the laser energy is higher than the one of the emitter. The fits yield $n = 1.09 \pm 0.16$ ($n = 1.21 \pm 0.08$) for red 5 (80) MHz and $n = 1.33 \pm 0.12$ for green 5 MHz excitation.

6 Lifetime measurement

To understand why our pulsed second–order correlation measurement looks like a cw measurement, we measured the lifetime of the excited state of emitter 1 with a lower repetition rate of 5 MHz compared to the second–order correlation measurement, and correlated the detected signal with the trigger from the laser in a standard time–correlated single–photon counting experiment. Figure S5 shows the data together with an double–exponential fit yielding a lifetime of 18.3 ± 1 ns and an additional small contribution of a fast decay of 0.7 ± 0.1ns. The fast decaying contribution increases with increasing excitation power and stems from a second decay channel.

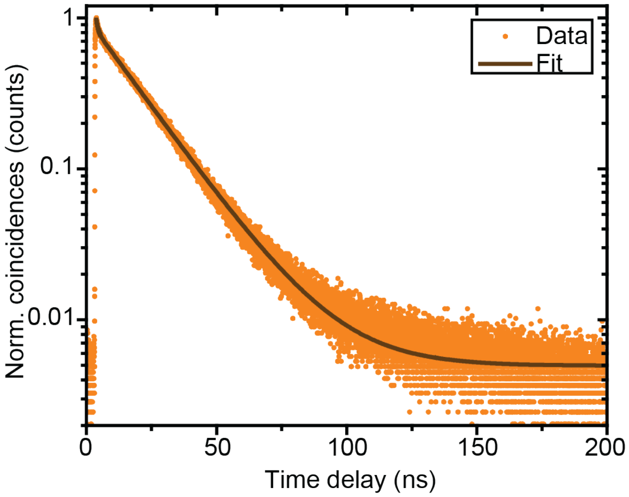

Figure S5: Lifetime measurement of emitter 1 under red 5 MHz excitation and fitted using a double–exponential curve.

7 Analysis of second–order autocorrelation measurements under non–resonant excitation

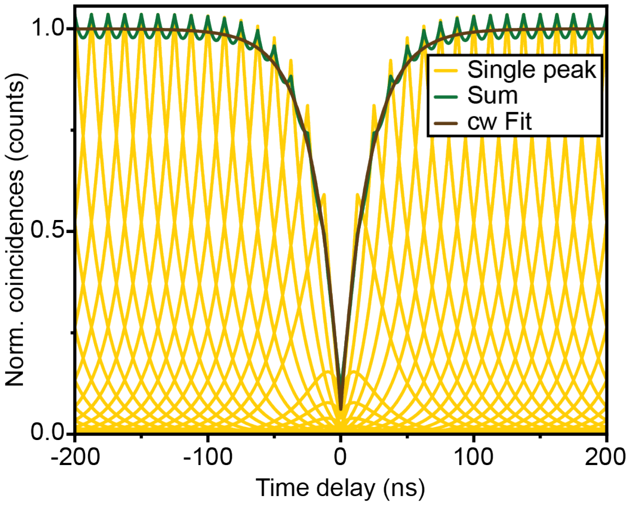

Figure S6: Simulation of a second–order correlation histogram for excitation with 80 MHz and an emitter lifetime of 18.3 ns.

The recorded time tag files were analyzed using ETA software[S3] with a binning of 2048 ps. To explain why our pulsed 80 MHz second–order correlation measurement resembles a measurement under continuous–wave (cw) excitation, we simulated the resulting histogram, which we show in Fig. S6. Single peaks from each excitation pulse with a repetition rate of 80 MHz with a lifetime of 18.3 ns are shown in yellow together with the resulting normalized histogram in green. The periodic modulations are not visible in our measurement in Fig. 3b and d due to noise. This strong overlap of the peaks justifies to treat the data

like a cw measurement and fitted with $g^{(2)}(\tau) = B\left(1 - \left(1 - g^{(2)}(0)\right)\right)\exp(-|\tau|/\tau_0)$ with the Poisson level B and the width τ_0, as demonstrated by the fit of the summed simulation peaks in brown.

The extracted width of the dip in the non–resonantly excited second–order autocorrelation measurement of emitter 1 is 6.69 ± 0.56 ns, which deviates from the measured lifetime using lower repetition rates, and can be explained by the high excitation power. The second–order correlation function can be described by $g^{(2)}(\tau) = 1 - \left(1 - g^{(2)}(0)\right)\exp(-|\tau|/(\tau_l + 1/W_p))$ as in Reference,[S4] with the emitter lifetime τ_l and the pump rate into the excited state W_p. This leads to a narrowing of the dip for high excitation powers, which was the case in our measurement (1.4 µW = 4.4 P_{sat}). This effect was not taken into account in the simplified simulation of the second–order autocorrelation measurement.

For the second–order autocorrelation measurement with 10 MHz repetition rate, the single peaks can be distinguished. Here, the non–postselected second–order coherence $g^{(2)}(0)$ is given by the ratio of summed up coincidences in the center peak and average number of coincidences in the side peaks. Since the peaks still overlap in time, well–defined time windows to sum up the coincidences cannot be given, and we analyzed the data following the procedure described in Reference.[S5]

8 Waveguide coupling simulations

We simulated the fabricated structure using a 3D-FDTD solver (Lumerical), and modeled the emitter as a dipole 5 nm above the 800 nm wide waveguide. The position of the dipole is swept across the width of the waveguide, and the directional coupling efficiency is shown in Fig. S7, where $x = 0$ nm represents the center of the waveguide, and $x = 400$ nm the edge. We swept the dipole across half of the waveguide width since the system is symmetric, and negative displacements will yield the same coupling conditions (i.e. one can mirror our results along the $x = 0$ nm line to obtain the full waveguide width sweep).

We average over the two dipole orientations parallel to the substrate (x and z in Fig. S7a). The directional coupling emission with the emitter at the edge of the waveguide is 0.3% (3.3%) to the TE00 (all) modes. The maximum simulated directional coupling efficiency into the TE00 (all) mode for averaged dipole orientation in–plane (x and z in Fig. S7a) was 2.5% (7.9%).

9 Methods for second–order autocorrelation measurements under resonant excitation

Figure S8 shows the modified optical setup for resonant excitation of the quantum emitter. For this measurement, we excited the emitter from the side with a 50 kHz linewidth continuous–wave diode laser by coupling it into the waveguide through a lensed fiber. The signal from the emitter as well as the remaining scattered laser light was collected by the microscope objective. To perform resonant excitation, the remaining laser has to be filtered with a polarization suppression setup. This requires well–defined excitation laser polarization, so that the remaining laser light is absorbed by a nanoparticle linear film polarizer

after emitter excitation, and only a fraction of the signal of the quantum emitter, with its polarization perpendicular to that of the laser, is detected. In our setup, the laser was further spatially suppressed using a free–space to fiber coupling, with the core acting as a pinhole. The filtered fiber-coupled signal was sent either onto the CCD of a spectrometer or into a Hanbury Brown an Twiss setup to perform second–order autocorrelation measurements. This part of the setup remained the same as for previous measurements. In our setup, the excitation was not perfectly polarized, since the lensed fiber inside the cryostat is not polarization maintaining and the laser was scattering out of the waveguide via the sidewall roughness. Nevertheless, the quarter– and halfwave plate (QWP, HWP) before the polarizer were aligned so that the remaining laser was minimized and on–the–fly optimization was possible during the measurements. The second–order autocorrelation measurement was recorded for a total amount of time of approx. 1.5 h, and required 3 realignments in addition to the on–the–fly polarization suppression optimization. The recorded timetag file was analyzed using readPTU[S6] with a time binning of 512 ns. The resulting histogram was fitted using the formula given above for cw autocorrelation measurements.

References

(S1) Castellanos-Gomez, A.; Buscema, M.; Molenaar, R.; Singh, V.; Janssen, L.; van der Zant, H. S. J.; Steele, G. A. Deterministic Transfer of Two-Dimensional Materials by All-Dry Viscoelastic Stamping. *2D Materials* **2014**, *1*, 011002.

(S2) Schell, A. W.; Tran, T. T.; Takashima, H.; Takeuchi, S.; Aharonovich, I. Non-Linear Excitation of Quantum Emitters in Hexagonal Boron Nitride Multiplayers. *APL Photonics* **2016**, *1*, 091302.

(S3) Extensible Timetag Analyzer. TIMETAG, 2019.

(S4) Michler, P.; Imamoğlu, A.; Mason, M. D.; Carson, P. J.; Strouse, G. F.; Buratto, S. K. Quantum Correlation among Photons from a Single Quantum Dot at Room Temperature. *Nature* **2000**, *406*, 968–970.

(S5) Schöll, E.; Hanschke, L.; Schweickert, L.; Zeuner, K. D.; Reindl, M.; Covre da Silva, S. F.; Lettner, T.; Trotta, R.; Finley, J. J.; Müller, K.; Rastelli, A.; Zwiller, V.; Jöns, K. D. Resonance Fluorescence of GaAs Quantum Dots with Near-Unity Photon Indistinguishability. *Nano Letters* **2019**, *19*, 2404–2410.

(S6) Ballesteros, G. C.; Proux, R.; Bonato, C.; Gerardot, B. D. readPTU: A Python Library to Analyse Time Tagged Time Resolved Data. *Journal of Instrumentation* **2019**, *14*, T06011–T06011.

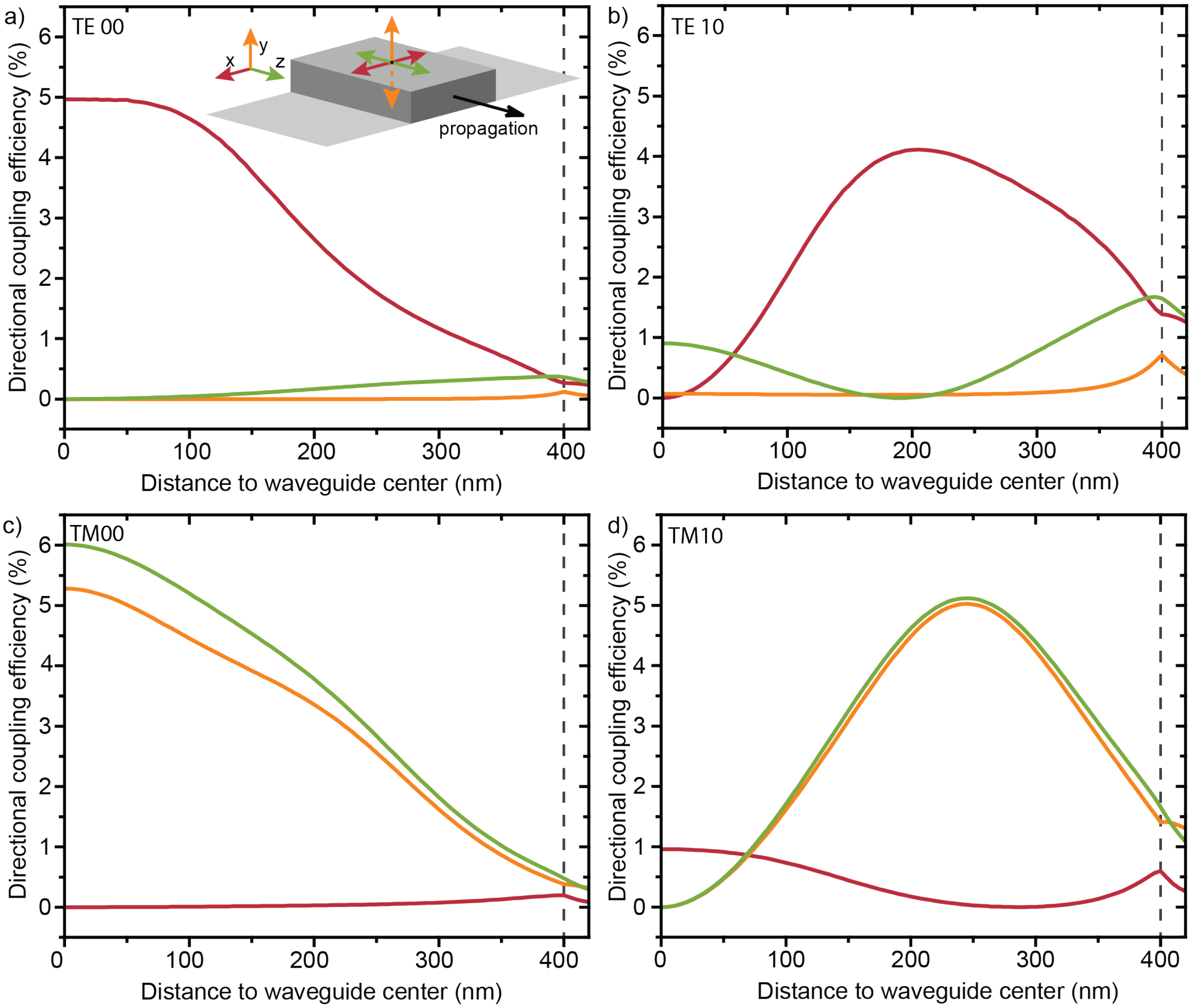

Figure S7: FDTD Simulation of the coupling efficiency to the guided modes (TE00, TE10, TM00, TM10) of the three different dipole orientations depending on the location on the waveguide. The dipole is located 5 nm above the waveguide. $x = 0$ nm is at the center of the waveguide.

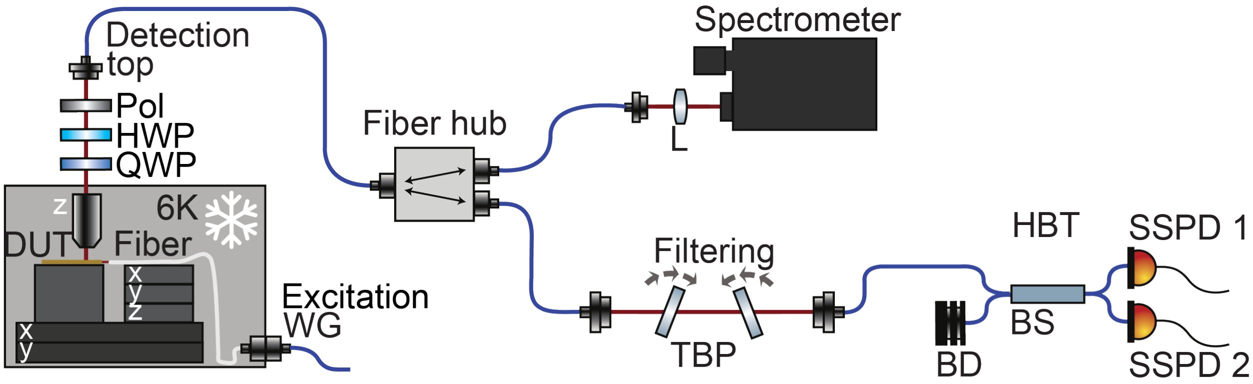

Figure S8: Side–excitation setup. The laser was coupled to the monolayer using a lensed fiber. The signal and scattered laser light was collected by the microscope objective, with the laser light being suppressed in a polarization suppression setup consisting of a quarter– (QWP) and half–wave plate (HWP) and a linear polarizer (Pol). The fiber–coupled filtered signal was routed in the fiber hub onto the spectrometer or the Hanbury Brown and Twiss setup (HBT), which included a free–space filtering by two tunable bandpass filters (TBP). DUT device under test; BS beam splitter; L lens; BD beam dump; SSPD superconducting single photon detector.
